# Promotion of wound healing using adipose-derived stem cells in radiation ulcer of a rat model

**DOI:** 10.1186/1423-0127-20-51

**Published:** 2013-07-22

**Authors:** Sheng-Ping Huang, Chun-Hsiang Huang, Jia-Fwu Shyu, Herng-Sheng Lee, Shyi-Gen Chen, James Yi-Hsin Chan, Shih-Ming Huang

**Affiliations:** 1 Graduate Institute of Life Sciences, National Defense Medical Center, Taipei 114, Taiwan; 2 Department of Biochemistry, National, Defense Medical Center, Taipei 114, Taiwan; 3 Department of Biology and Anatomy, National Defense Medical Center, Taipei 114, Taiwan; 4 Department of Pathology, Tri-Service General Hospital, National Defense Medical Center, Taipei 114, Taiwan; 5 Division of Plastic and Reconstructive Surgery, Department of Surgery, Tri-Service General Hospital, National Defense Medical Center, Taipei 114, Taiwan; 6 Department of Microbiology and Immunology, National Defense Medical Center, Taipei 114, Taiwan; 7 Department of Family and Community Medicine, Tri-Service General Hospital, National Defense Medical Center, Taipei 114, Taiwan

**Keywords:** Adipose-derived stem cells, Mesenchymal stem cells, Radiation ulcer, Wound model

## Abstract

**Background:**

Wound healing is a complex biologic process that involves the integration of inflammation, mitosis, angiogenesis, synthesis, and remodeling of the extracellular matrix. However, some wounds fail to heal properly and become chronic. Although some simulated chronic wound models have been established, an efficient approach to treat chronic wounds in animal models has not been determined. The aim of this study was to develop a modified rat model simulating the chronic wounds caused by clinical radiation ulcers and examine the treatment of chronic wounds with adipose-derived stem cells.

**Results:**

Sprague–Dawley rats were irradiated with an electron beam, and wounds were created. The rats received treatment with adipose-derived stem cells (ASCs), and a wound-healing assay was performed. The wound sizes after ASC treatment for 3 weeks were significantly smaller compared with the control condition (p < 0.01). Histological observations of the wound edge and immunoblot analysis of the re-epithelialization region both indicated that the treatment with ASCs was associated with the development of new blood vessels. Cell-tracking experiments showed that ASCs were colocalized with endothelial cell markers in ulcerated tissues.

**Conclusions:**

We established a modified rat model of radiation-induced wounds and demonstrated that ASCs accelerate wound-healing.

## Background

Approximately 50% of cancer patients receive radiation as an adjuvant therapy [1]. Although radiation therapy technology has progressed substantially, patients still suffer from various degrees of non-specific radiation damage to non-cancerous tissues [2]. Radiation ulcers have posed a formidable challenge to reconstructive surgeons [3,4]; they cause great distress to patients, impair their quality of life, and require enormous medical resources. These chronic wounds can last for several years, and some may even lead to amputation [5].

The mechanism of radiation injury is through damage to the DNA of cells, leading to cell apoptosis or necrosis. The histological features are vascular dilation and thrombosis, inflammation, and finally, hypovascular and fibrosis abscent of the epithelium and hair follicles. Radiation injury may cause redness, peeling, moist ulcers and other features depending on the severity. Ulcers may take several months to several years to heal [1].

Strategies for treating chronic wounds include the use of various growth factors [6-10], hyperbaric oxygen [11], and stem cells [7]. Mesenchymal stem cells (MSCs) have been considered to be suitable for application to ischemic wounds [12-14]. Mesenchymal stem cells may be derived from the bone marrow, umbilical cords or fat. Adipose-derived stem cells (ASCs) are easily isolated in large quantities because they are easy to culture and amplify, such that they have more potential for use in treatment applications than other cell preparations [15]. Extracts of uncultured fat applied to radiation skin ulcers and chronic lower extremity ulcers have been reported in clinical cases, and the wound-healing properties have been considered to be mediated by stem cells [16-18]. ASCs are considered to be suitable for the treatment of ischemic wounds because of their potential to differentiate into several cell types, such as endothelial cells, and secrete angiogenic and anti-apoptotic factors [19,20]. ASCs accelerate wound healing in ischemic models, such as diabetes-impaired wounds or mitomycin C-treated wounds [21,22]. The local delivery of ASCs via acellular matrix, such as an atelocollagen matrix, a silk fibroin-chitosan scaffold or acellular dermal matrix, has been considered a promising strategy to accelerate wound healing [22-24].

In this study, we attempted to establish a radiation-induced wound model and evaluate the effectiveness of *in vitro* cultured ASCs for the promotion of the wound healing of radiation ulcers via angiogenesis.

## Methods

### Animals

All of the animal experiments were approved and conducted under the guidance of the Institutional Animal Care and Use Committee (accredited by the Association for Assessment and Accreditation of Laboratory Animal Care International), National Defense Medical Center, Taipei, Taiwan.

### Isolation and culture of ASCs

Inguinal fat pads were harvested from 9- to 12-week-old Sprague–Dawley rats (BioLASCO, Taipei, Taiwan). The isolation and culture of ASCs were followed by our previous description [24]. The cells were used for experiments at passages 4–6.

### Mesenchymal stem cell immunophenotype

Cells from the fourth passage were analyzed for their immune phenotype. A total of 3 × 10^5^ cells were resuspended in 200 μl PBS containing 0.5% bovine serum albumin and incubated for 30 min at 4°C with monoclonal antibodies against rat CD29-fluorescein isothiocyanate (FITC) (BD Pharmingen, CA, USA), CD34-FITC (Santa Cruz Biotechnology, CA, USA), CD45-FITC (BD Pharmingen), CD90-FITC (BD Pharmingen), CD73 (mouse anti-rat; 5 F/B9, BD Pharmingen), CD31 (ab64543) or vWF (von Willebrand factor) (ab6994) (Abcam, Cambridge, UK) or the respective isotype control. For the detection of CD73, goat anti-mouse Alex Flour 488 (Invitrogen) was used for another round of incubation after PBS washing and resuspension in PBS containing 0.5% bovine serum albumin (Bio-Rad Laboratories, CA, USA). The cells were analyzed with a FACSCalibur cytometer (Becton Dickinson, NJ, USA) and this procedure has been described previously [24].

### ASC Differentiation Assays

Putative ASCs were incubated to differentiate into adipocytes and osteoblasts, described previously [24]. For adipocyte differentiation, cells reaching confluence were incubated for 7 repetitive cycles of 48 hours in adipogenic differentiation medium. For osteocyte differentiation, cells reaching confluence were cultured with osteogenic medium for 2 weeks. Oil red O (Sigma) and Alizarin red S (Sigma) dyes were used to identify adipocytes and osteoblasts, respectively.

### Real-time PCR

The total RNA of cultured cells at the indicated passage number was extracted with Tri Reagent (Ambion, TX, USA). cDNA was synthesized using Superscript III reverse transcriptase (Invitrogen, company location needed). Real-time PCR analysis was performed with a 7500 Real-time PCR system (Applied Biosystems, CA, USA) using Power SyBr Green PCR master mix (Applied Biosystems) with the following primers: VEGF sense, AGGCTGCACCCACGACAGAA; VEGF anti-sense, CTGGAAGATGTCCACCAGGG; HGF sense, TTTGGCCATGAATTTGACCTCT; HGF anti-sense, CTTCTGAACACTGAGGAATGTC; GAPDH sense, TCCTGCACCACCAACTGC; and GAPDH anti-sense, GCCATCCACAGTCTTCTG. GAPDH was used as an endogenous reference in each case. The data are presented as the average of triplicate experiments ± the standard error of the mean (SEM).

### ELISA assay

Rat ASCs isolated from three different rats were respectively cultured and expanded in culture medium (DMEM-10% FBS). At 70-90% confluence, cells were detached and passaged at a ratio of 1:3 at indicated passage numbers. After 24 hours incubation in culture medium, cells were switched to DMEM-5% FBS and incubated for 72 hours. Then, the condition medium was collected, and cell numbers were determined by trypan blue exclusion. The concentrations of vascular endothelial growth factor (VEGF) and hepatocyte growth factor (HGF) were measured using sandwich ELISA kit according to the manufacturer’s instruction: Rat VEGF Immunoassay and mouse/rat HGF Immunoassay kits were obtained from R&D Systems (Minneapolis, MN, USA). Data are presented as pictograms of the secreted factor per 10^4^ cells at the time of harvest.

### Wound model and wound-healing assay

Eight-week-old male rats weighing 250–300 g were used in this study. After anesthesia with Zoletil 50 (0.1 ml/100 g) (Virbac, Paris, France) and shaving the wool of the dorsum, four rats were irradiated with an electron beam (Primus, Erlangen, Germany) operated at 6 MeV. The dose rate was 900 cGy/min. A single dose of 50 Gy was given through an area of 3 cm diameter marked on the dorsal skin, and the surrounding healthy tissue was protected by a 1.2 cm lead shield. The assessment of acute skin reactions with a visual skin score began eleven days after radiation and was continued every two days until the 57th day after irradiation. The score was based on a previously described scoring method [25].

0 = normal;

0.25 = transient erythema, edema, or dry desquamation on dorsum or sole of the foot;

0.5 = slight epilation in <50% of exposure area;

1.0 = epilation in about 50% of exposure area;

1.5 = epilation in >50% of exposure area or some signs of dry desquamation on the leg; 2.0 = complete epilation, red foot, or dry desquamation in ≤50% of exposure area with epilation in ≥50% of exposure area;

2.5 = complete epilation with definite edema or dry desquamation in >50% area;

3.0 = moist desquamation in a small area; and

3.5 = moist desquamation in most of the area.

An additional three rats were irradiated with a 50 Gy electron beam, and the wound tissues were removed at the first, second and third weeks for histological observations of the wound model. Normal skin was removed from a healthy rat as a comparison.

To evaluate the wound size, the ulcerated area of each rat was recorded with a SANYO Xacti VPC-CA65 digital camera at weeks 3, 4, 5 and 6 post-radiation. Images were analyzed with Image-Pro Plus software. Discriminations between the ulcer and the re-epithelialization of the skin were performed by two investigators who were blind to group assignments.

### ASC transplantation and tracking

Three weeks after radiation, the rats were anesthetized, randomly divided into two groups (*n* = 8 each group), and injected with either 1 × 10^6^ ASCs resuspended in 0.8 ml of PBS (experiment group) or 0.8 ml PBS (control group) into the wound bed and margin. The two groups were treated with ASCs (experiment group) and PBS (control group) for four and five weeks post-radiation. The experiments ended at week 6 post-radiation, and the skin, including the wounds, was removed from each rat for histological examination, immunoblot analysis, vessel density assessment and dermal thickness measurement. Beside the animals used for the characterization of wound model and evaluation of ASCs treatment described previously, two additional rats were irradiated for tracing the transplanted ASCs in the ulcerated tissue described below.

The fourth-passage ASCs were labeled with 20 μM fluorescent cell tracker CM-DiI (chloromethyl-benzamido derivative of 1,1′-dioctadecyl-3,3,3′3′-tetra- methyl-inodcarbocyanine) (Molecular Probes, OR, USA) in accordance with the manufacturer’s recommendations. The procedure has been described previously [24].

### Histological examination and immunostaining

The removed skin, including the ulcers and re-epithelialized tissue, was fixed in 10% formalin for at least 24 h, embedded in paraffin, and sectioned in 5 μm increments. Frozen sections were prepared for the immunostaining of CD31 (ab64543) or vWF (ab6994) (Abcam, Cambridge, UK). For the staining of vWF, the tissues were fixed with 4% paraformaldehyde and permeabilized with 0.1% Triton X-100. For the staining of CD31, the tissues were fixed with 4% paraformaldehyde. Morphological observations were described previously [24].

### Immunoblot analysis

Skin samples were homogenized in lysis buffer (100 mM Tris–HCl, pH 8.0, 150 mM NaCl, 0.1% SDS, and 1% Triton 100) at 4°C. The cell extracts were separated through sodium dodecyl sulfate-polyacrylamide gel electrophoresis, transferred onto a polyvinylidene difluoride membrane (Millipore, MA, USA) and detected using antibodies against CD31 (TLD-3A12, BD Pharmingen) and Hu Antigen R (HuR) (3A2; Santa Cruz Biotechnology) as a control.

### Vessel density assessment

Three weeks after starting the treatment, wound tissues of ASC-treated or control group were removed and made into 8 μm frozen sections. After the staining of CD31 and DAPI, the blood vessel density of each wound was calculated and described as follows: for each wound (*n* = 8), five non-consecutive tissue sections were made. For each section, three randomly selected fields (400× magnification) of ulcer tissues were photographed by fluorescence microscopy (Leica, Wetzlar, Germany). A total of 15 images were taken for each wound, and neo-vasculation was assessed by measuring the CD31 stained capillaries.

### Dermal thickness measurement

Dermal thickness was measured in eight animals from each group and normal skin as the control. A representative image of H&E-stained sections was obtained. The dermal thickness was measured at three points in each image.

### Statistical analysis

All of the data were calculated as the mean ± SEM. Statistical analyses were performed using a two-tailed Student *t* test. Significant differences were defined by *p* < 0.05.

## Results

### Characterization of rat ASCs

**Figure 1:**
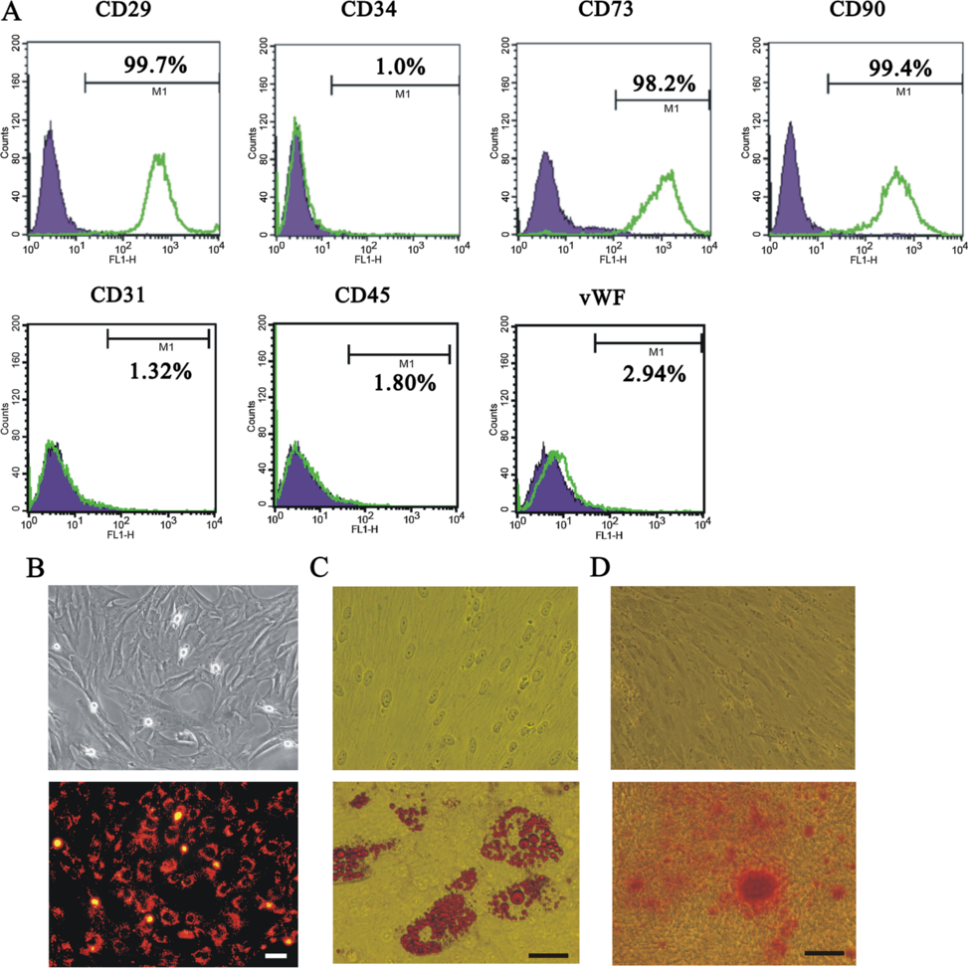
**Identification of rat ASCs. (A)** The 4th passage of rat ASCs was analyzed with flow cytometry for the expression of CD29, CD31, CD34, CD45, CD73, CD90 and vWF biomarkers. Green lines represent the specific fluorescent antibodies, and black lines represent isotype controls. **(B)** The 4th passage of rat ASCs was labeled with CM-DiI and observed under a fluorescence microscope. Bar: 50 μm. ASCs were assessed for adipogenic and osteogenic differentiation, and the cells were stained with Oil red O **(C)** and Alizarin red S **(D)**, respectively. Scale bar, 200 μm.

**Figure 2:**
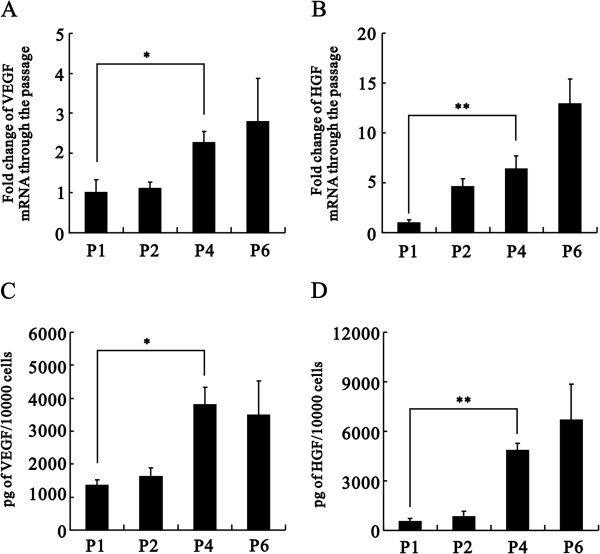
**Effects of passage number on mRNA expression and secretion of VEGF and HGF.** Passage numbers were shown as P1, P2, etc. **(A-D)** Total mRNAs were extracted from three different rat ASCs and reverse transcribed into cDNA. The VEGF **(A)** and HGF **(B)** mRNA expression levels were analyzed using real-time PCR analysis. Values were normalized to the level of GAPDH mRNA and expressed relative to normalized values of P1. Secretion of VEGF **(C)** and HGF **(D)** by rat ASCs (*n* = 3) cultured over 72 hours was measured by ELISA. The data represent the mean ± SEM. *p < 0.05, **p < 0.01.

First, we characterized specific cell surface markers of our isolated cells with flow cytometry analysis. Our fourth generation of isolated cells was positive for CD29, CD73 and CD90 but little evidence of hematopoietic lineage markers CD34 and CD45 and endothelial cell markers CD31 and vWF, (Figure 1A), consistent with the characterization of ASCs. The growth of our cultured ASCs was similar to that of the fibroblast-like phenotype (Figure 1B). ASCs were labeled with the cell tracker CM-DiI for the cell-tracking experiment described later. In addition, our cultured ASCs had the ability to differentiate into adipocytes and osteocytes, which were verified with Oil-Red O and Alizarin Red S staining, respectively (Figure 1C and D). These results suggested that our isolated cells had characteristics consistent with MSCs [19]. We further examined the mRNAs, and secretory proteins of VEGF and HGF, two well-known angiogenic factors, in different rat sources used for isolating ASCs (Figure 2). Mostly constant expressions of VEGF and HGF mRNAs in real-time PCR analysis and secretory proteins in ELISA analysis were observed at every passage considered, and significant changes were found at the 4th passage of both factors (Figure 2), suggesting, at least in part, secretory factors might play the functional roles for the ASC effect. Hence, we used 4th passage of ASCs for further experiments in this study.

### The establishment of a wound model of acute radiation skin ulcers

**Figure 3:**
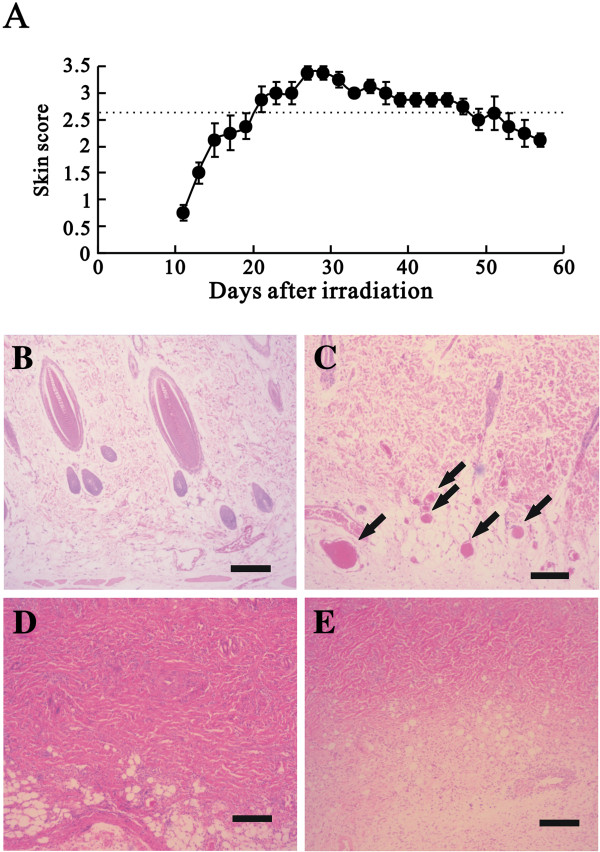
**Wound model of an acute radiation ulcer.** The animal model of radiation ulcers and the experimental design are described in the Materials and Methods. **(A)** The skin scores of rats were evaluated after 50 Gy electron irradiation (*n* = 4). **(B-E)** Histological examination was performed by hematoxylin and eosin staining of the skin reaction. The histology of skin exposed to 50 Gy irradiation after 0 days (normal skin) **(B)**, 7 days **(C)**, 14 days **(D)** and 21 days **(E)** is shown. Arrow: vascular dilation and thrombosis. Bar: 200 μm.

Male Sprague–Dawley rats were exposed to the indicated radiation on the dorsal skin. We assessed radiation injuries with skin scores (from 0 to 3.5, detailed score information shown in the Method section) every two days from 11 to 57 days after exposure, and the average score was 2.63 ± 0.08 (Figure 3A, dashed line). Peeling in the radiated areas was observed approximately two weeks after radiation exposure. The most serious skin damage, moist ulcers in the exposed areas, was observed four weeks after radiation. The festering wounds had still not fully re-epithelialized at the eighth week after exposure. The ulcerated areas achieved their maximum size at approximately the third week (data not shown), and this time point was used as the starting point for ASC treatment in subsequent experiments.

Histological observation showed the characteristics of radiation ulcers on skin. The dermis of normal skin on the control rats contained hair follicles (Figure 3B). One week (score = 0) after irradiation, vascular dilution and thrombosis were clearly observed (Figure 3C, indicated as arrows), although the epidermis had not yet fallen off. Day14 (score around 2.5) and day 21 (score around 3) after irradiation, fibrosis and a lack of epithelium and follicles in the tissue were observed (Figure 3D and E) as previously described [3]. More importantly, the weight of all of the rats continued to increase after radiation (data not shown), suggesting that radiation injury did not cause life-threatening stress to the rats. Therefore, we had established a model of acute skin radiation injury.

### Transplantation of ASCs could enhance wound healing

**Figure 4:**
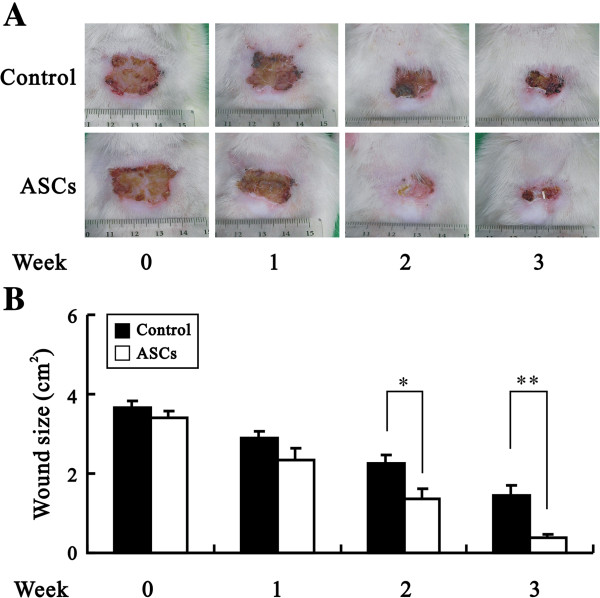
**ASCs promoted the wound healing of skin with radiation ulcers.** Radiation ulcers were induced and then treated with the indicated ASCs. **(A)** Representative images of wounds are shown at weeks 0, 1, 2 and 3 from eight rats in each group. **(B)** The wound size of the control (PBS treated; black bar) and ASC-treated (white bar) rats was measured. The data represent the mean ± SEM of eight rats. *p < 0.05; **p < 0.01.

ASCs were used to treat skin wound areas of approximately 3.5 cm^2^ three weeks after irradiation (Figure 4A or 4B were not labeled). To allow for even distribution within the wounds, ASCs were equally divided and injected into 12 locations in the wound bed and edge. The wound sizes in the ASC-treated and control groups were recorded every week. Our quantitative data were significantly different at the second and third weeks after starting treatment (week 2, * *p* < 0.05; week 3, ** p < 0.01) (Figure 4B), indicating the ASC treatment enhanced the healing of radiation ulcers.

### Transplantation of ASCs increased angiogenesis and granulation in wound healing

**Figure 5:**
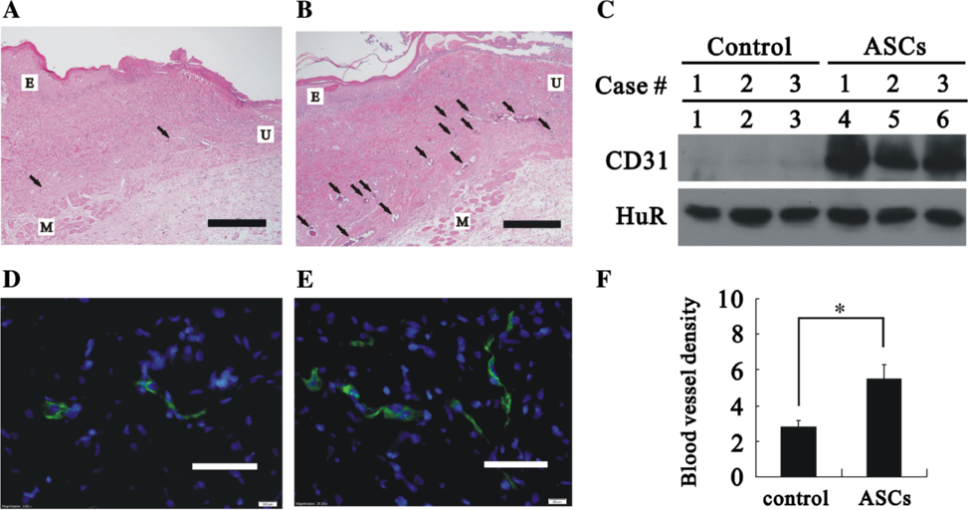
**Analysis of angiogenesis and blood vessel density in ASC-treated tissue. (A-C)** After three weeks of treatment, the tissues of the wound edge were subjected to histological examination. Compared with the control group **(A)**, the capillary density in the dermis was clearly increased in the ASC-treated group **(B)**. Arrows indicate vessel formation and proliferation sites containing erythrocytes in each wound. E: epidermis; M: muscle layer; U: ulcer. Bar: 500 μm.** (C)** Tissue lysates from re-epithelialized skin tissue of control and ASC-treated groups were analyzed with immunoblot analysis against CD31 and HuR (a loading control). **(D-F) **Three weeks after ASC treatment, ulcerated tissues were removed and processed into 8 μm frozen sections. Slides were stained with CD31 (green) and DAPI, a fluorescent nuclear stain (blue). Bar: 50 μm. Representative images of the control **(D)** and ASC **(E)** group are shown. **(F)** The blood vessel density was evaluated in the control and ASC-treated groups. The data represent the mean ± SEM of eight rats. *p < 0.01.

**Figure 6:**
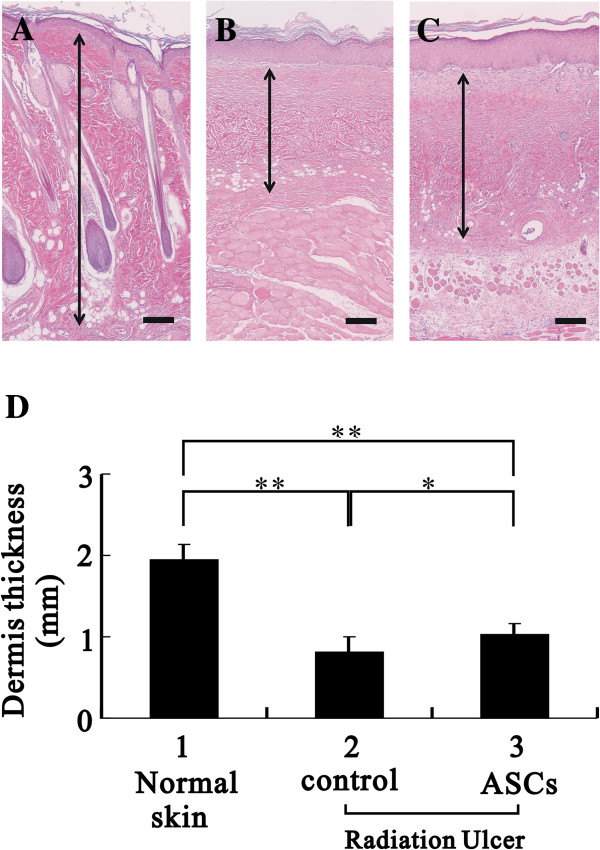
**Histological assessment demonstrated that ASCs significantly improved the granulation rate. (A)** The dermis of normal skin served as a comparison. After three weeks of ASC treatment, the healed skin of each group was assessed. The images are representative of the control **(B)** and ASC-treated **(C)** groups. Bar: 200 μm. **(D)** The thickness of the dermis was evaluated. The data represent the mean ± SEM of eight rats. *p < 0.05; **p < 0.001.

After the third week of ASC treatment, tissues from the wound edge and healing region were examined with histological examination and immunoblot analysis (Figure 5). Tissue from the wound edge (labeled as U) showed growth of the epithelium (labeled as E) and muscle (labeled as M) toward the ulcer region, and tissue between the epithelial and muscle layers showed dermal fibrosis with a lack of hair follicles (Figure 5A and B). The capillary density in the dermis was clearly increased in the ASC-treated group compared with the control group (Figure 5A and B, indicated by arrows). The re-epithelialization of healing skin was identified by a lack of hair. In the healing regions, the average CD31 (a vascular endothelial cell marker) expression of the ASC-treated group was significantly higher than the control group with immunoblot analysis (Figure 5C). ASCs also significantly increased blood vessel density (Figure 5D-E) and the dermal thickness of the healed skin (Figure 6A-C) in ulcerated tissues. Our results suggested that ASCs significantly increased the dermal thickness compared with control, but the thickness was significant low than normal skin (Figure 6D). Taken together, these results indicate that the treatment with ASCs could increase the blood supply and the granulation rate in wound healing compared to control condition.

### Tracking of CM-DiI-labeled ASCs after transplantation

**Figure 7:**
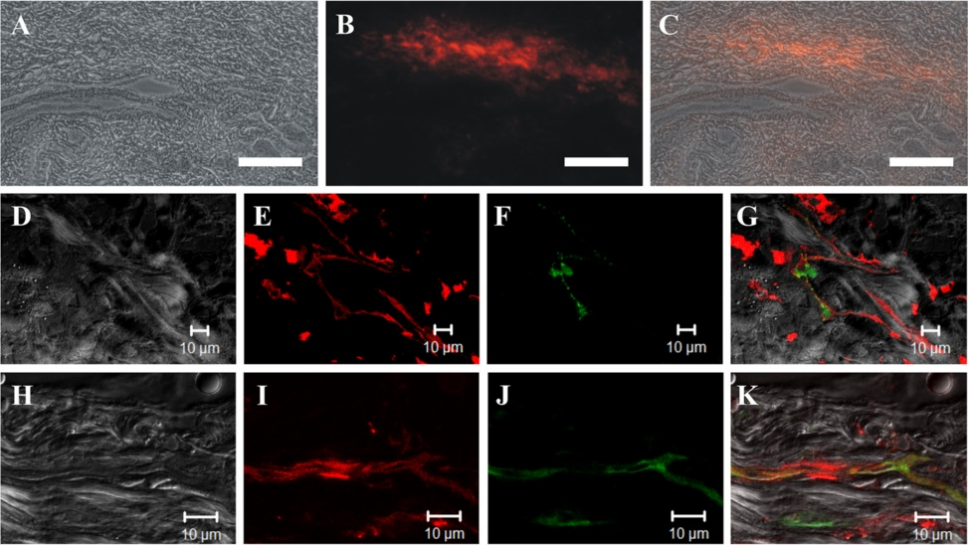
**Engrafted CM-DiI-labeled ASCs were expressed within the endothelial cells. (A-C)** Images obtained from 16 μm sections frozen 14 days after the injection of CM-DiI-labeled ASCs (red fluorescence) into tissue with radiation ulcers. Phase contrast **(A)**, red fluorescence **(B)** and overlay images **(C)**. Bar: 200 μm. **(D-K)** Confocal images were acquired from 8 μm sections frozen 14 days after the injection of CM-DiI-labeled ASCs and immunostained with vWF **(D-G)** or CD31 **(H-K)**. Differential interference contrast image **(D and H)**. Red staining indicates CM-DiI-labeled ASCs **(E and I)**. Green staining indicates vWF **(F)** and CD31 **(J)**. Overlay image **(G and K)**.

To identify the survival, distribution and differentiation characteristics of ASCs after transplantation, we injected CM-DiI-labeled ASCs into the ulcerated tissue and analyzed the preparations two weeks later. In frozen sections, CM-DiI-labeled cells were clearly distinguished in the ulcerated tissue; these cells clustered at the site of the injection but did not disperse into the tissue (Figure 7A–C). The nuclei of CM-DiI-labeled cells were labeled with DAPI, and parts of these nuclei colocalized with the cell proliferation marker Ki67, implying that allografts of ASCs could allow for the survival and proliferation of the tissue of the radiation ulcer (data not shown). In addition, a portion of CM-DiI-labeled cells colocalized with the vascular endothelial cell markers vWF (Figure 7D-G) and CD31 (Figure 7H-K) and showed a dendritic vascular structure, indicating that ASCs can contribute to newly formed vasculature in the ulcerated tissue. The punctate staining of vWF in Figure 7F suggests that the vWF stored in Weibel-Palade bodies of endothelial cells is consistent with literature descriptions [26,27].

## Discussion

As an adjuvant therapy for cancer, Siemens Primus produces electron or photon radiation for use on the surface layer of the skin or for deep tissue damage, respectively. In this study, we used electron beam radiation to simulate skin damage during cancer treatment. Approximately one week later, the skin began to deteriorate in appearance; the wound gradually showed edema and epilation and eventually showed moist desquamation in the exposure area (Figure 3, days 14–21). These histological observations are consistent with the characteristics of radiation ulcers. Therefore, we did develop a modified rat model of acute radiation skin damage for chronic wounds in this study. In contrast with previous models (combined radiation–wound or leg ulcers caused by radiation) [28,29], our model more closely simulates the chronic wounds caused by clinical radiation ulcers and can provide more adequate tissue for observation and biochemical analysis.

Isolated ASCs may contain several cell types, including endothelial cells, smooth muscle cells, pericytes, fibroblasts, and preadipocytes [30]. With the increase in passage numbers, cells gradually present a more homogeneous cell population [31]. Although ASCs can differentiate into various cell types, such as adipocytes and osteocytes in this study, these capabilities may vary with the cell culture environment and passage number. For example, studies have shown that ASCs can differentiate into myocardial cells in primary cell culture but not in later generations of cells [32]. To achieve the best applications for different purposes, culture conditions, passage number and other factors that may affect cell ability are worthy of more detailed study in the future. In this study, the results of real-time PCR and ELISA analysis indicated that the mRNA and secretory protein expressions of VEGF and HGF in ASCs showed an increasing trend with increasing passage number. The expression level reached a peak at approximately the 4th-6th generations of ASCs. Therefore, we chose the 4th generation of ASCs for subsequent experiments. It is a puzzle to demonstrate paracrine effects and/or direct ASC effects, however, both effects are supported by previous studies [20,21].

Clinical cases of the application of processed lipoaspirate (PLA) cells in radiation ulcer treatment indicate that isolated ASCs within PLA cells may play a key role in wound healing [18]. We further demonstrated that the allogeneic transplantation of *in vitro* cultured ASCs enhanced the healing of radiation ulcers. The results of cell-tracking experiments, histological examination, and immunoblot analysis indicate that ASCs could increase the blood supply and granulation rate in wound healing. In addition, cell-tracking experiments also showed that ASCs could survive (14 days) and proliferate in the ulcers (data not shown), suggesting that these cells create long-term effects in ulcers. Consistent with previous studies, our results indicate that transplanted ASCs colocalized with endothelial markers [24,33]. However, these data are not enough to conclude that ASCs differentiated into endothelial cells because the colocalization of these signals may be due to cell fusion between transplanted ASCs and endothelial cells of the host. To address this question, heterograft experiments combined with species-specific antibody examination should be carried out in the future.

Strategies for treating chronic wounds include various growth factors [6-10], hyperbaric oxygen [11], and stem cells [8,10]. MSCs have been considered suitable for application to ischemic wounds [12-14]. MSCs may come from bone marrow, umbilical cords or fat. ASCs are easily isolated in large quantities, being easy to culture and amplify, and therefore have more potential for treatment applications [15]. Extracts of uncultured fat applied to radiation skin ulcers and chronic lower-extremity ulcers have been reported in clinical cases, and it is considered that the wound-healing properties come from stem cells [16-18]. ASCs are considered suitable for the treatment of ischemic wounds because of their potential for secretion of angiogenic and anti-apoptotic factors, and differentiation into several cell types such as endothelial cells [19,20]. ASCs accelerate wound healing in ischemic models such as diabetes-impaired wounds or mitomycin C-treated wounds have been reported [21,22]. Local delivery of ASCs via acellular matrix such as silk fibroin-chitosan scaffold, atelocollagen matrixwas or acellular dermal matrix has been considered a promising strategy to accelerated wound healing [22-24]. Previous studies have shown that bone marrow-derived MSCs that express human platelet-derived growth factor A and human beta-defensin 2 promote wound healing [28], and such a strategy may apply to our ASC treatment of radiation ulcers.

In the future, more effort will be required to improve the use of ASCs in the treatment of radiation injuries. In this study, we injected ASCs into the site of the radiation wound. However, cells injected into the wound bed did not spread evenly but clustered at the location of the injection. To overcome this obstacle, ASC therapy through ASC-seeded scaffolds [15,34] or monolayer stem cells [32] may be applied. In future clinical applications, patients with local radiation injuries may use ASCs from non-irradiated sites and those with systemic radiation injuries may use ASCs or adipose tissue from ‘stockpiles’ or ‘banks’ for treatment [35].

## Conclusions

We established a rat model of acute radiation ulcers and demonstrated that the allograft transplantation of *in vitro* cultured ASCs improved the wound healing of radiation ulcers. The histological examination of the wound edge and the immunoblot analysis of the re-epithelialization region both indicated that ASCs contributed directly to vascularization by acting as angiogenesis-promoting cells and/or likely by differentiating into endothelial cells. In the future, a better understanding of the impact of ASCs on ulcers and improvements in the technology of ASC treatment and storage will contribute to more effective ASC therapy for radiation ulcers and other chronic wounds.

## Abbreviations

MSCs: Mesenchymal stem cells; ASCs: Adipose-derived stem cells; FITC: Fluorescein isothiocyanate; vWF: von Willebrand factor; VEGF: Vascular endothelial growth factor; HGF: Hepatocyte growth factor; CM-DiI: Chloromethyl-benzamido derivative of 1,1′-dioctadecyl-3,3,3′3′-tetra- methyl-inodcarbocyanine.

## Competing interests

The authors declare that they have no competing interests related to this work.

## Authors’ contributions

SPH carried out the preparation of stem cell and culture, PCR, ELISA, immunostaining analysis, wound healing model and drafted the manuscript. CHH carried out the preparation of stem cell, culture, PCR and immunoblotting analysis. JFS participated in the immunostaining and trafficking analysis. HSL participated in the histologic examination. SGC, JYHC and SMH conceived of the study, and participated in its design and coordination and helped to draft the manuscript. All authors read and approved the final manuscript.
